# Serum retinol binding protein 4 is associated with visceral fat in human with nonalcoholic fatty liver disease without known diabetes: a cross-sectional study

**DOI:** 10.1186/s12944-015-0033-2

**Published:** 2015-04-16

**Authors:** Xinxia Chang, Hongmei Yan, Hua Bian, Mingfeng Xia, Linshan Zhang, Jian Gao, Xin Gao

**Affiliations:** Department of Endocrinology and Metabolism, Zhongshan Hospital, Fudan University, Shanghai, 200032 China; Department of clinical nutrition, Zhongshan Hospital, Fudan University, Shanghai, 200032 China

**Keywords:** Retinol binding protein 4, Visceral abdominal fat, Non-alcoholic fatty liver disease, Hepatic fat content

## Abstract

**Background:**

High serum Retinol Binding Protein 4 (RBP4) levels were associated with insulin-resistant states in humans. To determine which fat compartments are associated with elevated RBP4 levels in humans, we measured serum RBP4 and hepatic fat content (HFC), visceral (VFA) and subcutaneous abdominal fat area (SFA) in 106 subjects with non-alcoholic fatty liver disease (NAFLD) without known diabetes.

**Methods:**

106 patients with NAFLD (M/F: 61/45, aged 47.44 ± 14.16 years) were enrolled. Subjects with known diabetes, chronic virus hepatitis, and those with alcohol consumption ≥30 g/d in man and ≥20 g/d in woman were excluded. Anthropometrics and laboratory tests, including lipid profile, alanine aminotransferase (ALT), aspartate aminotransferase (AST) and γ-glutamyltransferase (γ-GT) were conducted. HFC, VFA and SFA were determined by CT scan. Serum RBP4 was detected by an enzyme immunoassay kit and validated by quantitative Western blotting.

**Results:**

Circulating RBP4 was negatively associated with high-density lipoprotein cholesterol (HDL-c) (r = −0.392, *p* < 0.001), but positively with waist-to-hip ratio (WHR) (r = 0.343, *p* = 0.001), triglyceride (r = 0.330, *p* = 0.002), VFA (r = 0.298, *p* = 0.027), systolic blood pressure (r = 0.247, *p* = 0.020), diastolic blood pressure (r = 0.241, *p* = 0.023), γ-GT (r = 0.239, *p* = 0.034), waist circumference (r = 0.218, *p* = 0.040). Differently, serum RBP4 levels were not associated with HFC (r = 0.199, p = 0.071), SFA, age, BMI, total cholesterol, low-density lipoprotein cholesterol (LDL-c), ALT or AST (all *p* > 0.05). Multiple linear regression analysis revealed that RBP4 correlated independently with VFA (Standard β = 0.357, *p* = 0.019) and HDL-c (Standard β = −0.345, *p* = 0.023) in all subjects, HDL-c (Standard β = −0.315, *p* = 0.040) in men, VFA/SFA in women (Standard β = 0.471, *p* = 0.049), not with HFC. However, serum RBP4 was positively correlated with HFC when HFC below 6.34% (r = 0.574, *p* = 0.001).

**Conclusions:**

RBP4 could be a marker of abdominal obesity, however, the role of RBP4 in the pathogenesis of NAFLD is not sufficiently elucidated.

## Background

Retinol binding protein 4(RBP4) is a 21 kDa secreted protein that is the sole specific transport protein for vitamin A (retinol) in blood [1]. RBP4 belongs to the lipocalin family of proteins that transport small hydrophobic molecules [2]. The RBP4 gene is located on chromosome 10 (10q23–q24) near the region that has been linked to increased fasting glucose levels in European Caucasians and to type 2 diabetes in Mexican–Americans [3,4]. Liver has the highest expression level of RBP4; however, adipose tissue has the second highest rate of expression [5]. In adipose tissue, RBP4 expressed predominately in mature, lipid-laden adipocytes [6].

Studies in mice suggest that elevated serum RBP4 could play a causal role in insulin resistance. Increasing serum RBP4 concentrations causes insulin resistance, by transgenic overexpression or by injection of purified RBP4 protein into wild-type mice [7]. Conversely, RBP4 knockout mice exhibit enhanced insulin sensitivity [7]. Serum RBP4 concentrations are also elevated in insulin-resistant humans, including those who suffer from obesity, impaired glucose metabolism [7-9]. A number of studies in humans show that serum RBP4 levels correlate inversely with insulin sensitivity and/or positively with many other components of the metabolic syndrome [8,10-17]. Improving insulin sensitivity by interventions such as lifestyle modification, insulin-sensitizing drug rosiglitazone or gastric banding surgery, is associated with lowering of serum RBP4 in humans [8,13,14,17].

Abdominal obesity is one of the main characteristics of the insulin resistance. Some studies have observed the relationship between RBP4 and abdominal obesity and found that serum RBP4 concentrations in humans correlate highly with waist-to-hip circumference ratio [8], waist circumference [11], and percent trunk fat [15]. Nora Kloting [18] found that serum RBP4 correlated positively with adiposeRBP4 mRNA and intra-abdominal fat mass and inversely with insulin sensitivity, independently of age, gender, and body mass index. Therefore, visceral fat may be a major source of RBP4 in insulin-resistant states.

So, we speculate that nonalcoholic fatty liver disease (NAFLD), a typical case of visceral obesity, is associate more closely with RBP4. However, research conclusions about the relationship of RBP4 and hepatic fat content are controversial. Some researches indicated that RBP4 is elevated in non-diabetic NAFLD patients [9], or diabetic NAFLD [19] or morbidly obese women, specifically, in morbidly obese subjects with NAFLD [20], and selected studies claimed that there exists a positive association between RBP4 and liver enzymes, specifically ALT and GGT [9].Circulating RBP-4 correlated positively with liver fat which may reflect its effects on hepatic insulin sensitivity [21]. In contrast, some researches demonstrated that serum RBP4 is not a predictive factor in NAFLD [22], and RBP4 did not differ among NAFLD histological groups and thus did not identify non-alcoholic steatohepatitis (NASH), the advanced stage of NAFLD [23], another study showed that in NAFLD patients, serum RBP4 was significantly lower as compared with controls and did not correlate with insulin resistance [24]. Up to now, the relationship of RBP4 and hepatic fat content (HFC) is still controversial, and the relevant data of Chinese is blank.

Thus, the aim of this paper was to investigate the association between serum RBP4 levels and HFC, visceral (VFA) and subcutaneous abdominal fat area (SFA), as well as other metabolic parameters in Chinese subjects with nonalcoholic fatty liver disease without known diabetes.

## Results

### The general characteristics of the subjects

HFC of all the subjects determined by CT scan varied from 0.56% to 24.7% with a mean and standardized deviation of 7.9% and 6.9%, respectively. By dividing the distribution of HFC into quartile, we found that there were more women subjects than men subjects in groups with lower HFC (Q1), but in groups with higher HFC (from Q2 to Q4) there were more men subjects (Table 1).

**Table 1:** **Clinical and laboratory features of the subjects**

**Liver fat content (HFC)**	**Q1 HFC < 3.26% (n = 26)**	**Q2 HFC: 3.26% ~ 6.33% (n = 27)**	**Q3 HFC: 6.34% ~ 10.64% (n = 27)**	**Q4 HFC ≥10.65% (n = 26)**	***P*** **value**
Sex (M/F)	9/17	16/11	19/8	17/9	0.043
Age (years)	47.15 ± 13.91	44.96 ± 12.18	50.30 ± 13.09	46.00 ± 16.17	0.489
BMI (kg/m^2^)	24.79 ± 2.90	25.64 ± 3.89	27.74 ± 2.94^▲▼^	28.58 ± 2.81^▲▼^	<0.001
Waist(cm)	84.86 ± 9.09	89.20 ± 9.60	94.43 ± 6.19^▲▼^	99.02 ± 9.26^▲▼^	<0.001
WHR (cm/cm)	0.88 ± 0.08	0.92 ± 0.07^▲^	0.95 ± 0.05^▲^	0.95 ± 0.07^▲^	0.001
SBP (mmHg)	121.12 ± 19.01	123.63 ± 13.69	132.63 ± 18.62^▲^	138.88 ± 21.90^▲▼^	0.002
DBP (mmHg)	75.88 ± 11.92	79.22 ± 10.08	81.96 ± 8.91^▲^	88.81 ± 10.35^▲▼◆^	<0.001
TC (mmol/L)	4.78 ± 0.92	4.99 ± 0.87	4.91 ± 0.68	5.22 ± 0.86	0.308
TG (mmol/L)	1.62 ± 0.82	2.27 ± 1.31^▲^	2.20 ± 1.26^▲^	2.50 ± 1.31^▲^	0.023
HDL-c (mmol/L)	1.30 ± 0.30	1.16 ± 0.24	1.08 ± 0.21^▲^	1.10 ± 0.21^▲^	0.009
LDL-c (mmol/L)	2.72 ± 0.73	2.99 ± 0.62	2.81 ± 0.58	3.04 ± 0.79	0.305
ALT (U/L)*	16.0(4.0-117.0)	28.0(11.0-124.0)^▲^	38.0(12.0-121.0)^▲^	63.0(11.0-259.0)^▲▼◆^	<0.001
AST (U/L)*	20.0(11.0-50.0)	23.0(11.0-78.0)	29.0(11.0-82.0)^▲^	45.0(14.0-111.0)^▲▼◆^	<0.001
ALP (U/L)	64.22 ± 16.93	74.17 ± 17.55	76.21 ± 26.67	77.29 ± 18.96^▲^	0.121
γ-GT (U/L)*	20.0(8–118.0)	32.5(15.0-139.0)^▲^	38.5(17.0-112.0)^▲^	67.0(16.0-378)^▲▼◆^	<0.001
VFA (cm^2^)	98.12 ± 32.84	105.21 ± 36.02	121.48 ± 35.74	134.14 ± 31.11^▲▼^	0.040
SFA (cm^2^)	86.91 ± 22.81	76.51 ± 34.99	87.51 ± 27.61	109.88 ± 35.62^▼^	0.032
VFA/SFA	1.15 ± 0.35	1.59 ± 0.77	1.62 ± 0.97	1.35 ± 0.52	0.374
Serum RBP4 (μg/ml)	43.23 ± 1.19	54.00 ± 8.74	51.75 ± 1.52	54.68 ± 8.86	0.298

Data are presented as means ± S.D or median (range).

*These variables were log transformed before analyses.

▲: compared with group Q1, P < 0.05; ▼: compared with group Q2, P < 0.05; ♦: compared with group Q3, P < 0.05.

Accompanied with the increase of HFC, BMI, waist circumference, WHR, systolic blood Pressure (SBP), diastolic blood pressure (DBP), triglyceride (TG), alanine aminotransferase (ALT), aspartate aminotransferase (AST), γ-glutamyltransferase (γ-GT), VFA and SFA elevated gradually, high-density lipoprotein cholesterol (HDL-c)fell gradually (both *p* < 0.05). The four groups did not differ in some metabolic parameters, including age, total cholesterol (TC), low-density lipoprotein cholesterol (LDL-c), alkaline phosphatase (ALP). No significant differ were found in some metabolic parameters in the four groups, including age, total cholesterol (TC),low-density lipoprotein cholesterol (LDL-c), alkaline phosphatase (ALP).

### Serum RBP4 and VFA, SFA and other metabolic parameters

In all the subjects, serum RBP4 levels were significantly associated with sex, weight, waist circumference, WHR, SBP, DBP, TG, HDL-c, γ-GT, VFA, VFA/SFA, but not associated with age, BMI, TC, LDL-c, ALT, AST, and ALP (Table 2). Considering RBP4 is sex related, we did the correlation analysis respectively according to gender. In women, serum RBP4 level was only significantly associated with VFA/SFA, but not associated with HFC, BMI, liver enzymes and the other variables mentioned above. While in men, serum RBP4 level was significantly associated with HDL-c, ALT, AST, but not associated with HFC, VFA, SFA, BMI and the other variables mentioned above (Table 2).

**Table 2:** **Univariate correlation of serum RBP4 in all the subjects**

	**Total (n = 106)**		**Men (n = 61)**		**Women (n = 45)**	
	**r**	***P***	**r**	***P***	**r**	***P***
Sex	−0.415	<0.001				
Age (y)	0.061	0.574	0.114	0.432	0.252	0.126
Weight (kg)	0.245	0.021	0.06	0.674	0.003	0.986
BMI (kg/m^2^)	0.048	0.653	0.034	0.814	−0.018	0.915
Waist (cm)	0.218	0.040	0.069	0.632	0.121	0.468
WHR	0.343	0.001	0.139	0.332	0.264	0.109
SBP (mmHg)	0.247	0.020	0.138	0.332	0.299	0.068
DBP (mmHg)	0.241	0.023	0.196	0.167	0.124	0.458
TC (mmol/L)	0.095	0.382	0.175	0.228	0.076	0.652
TG (mmol/L)	0.330	0.002	0.124	0.398	0.188	0.257
HDL-c (mmol/L)	−0.392	<0.001	−0.298	0.045	−0.183	0.279
LDL-c (mmol/L)	0.049	0.658	0.108	0.476	0.113	0.504
ALT (U/L)	0.105	0.335	−0.287	0.048	0.168	0.312
AST (U/L)	−0.077	0.487	−0.304	0.038	0.055	0.752
ALP (U/L)	0.116	0.310	−0.007	0.962	0.171	0.326
γ-GT (U/L)	0.239	0.034	0.142	0.357	0.042	0.810
SFA (cm^2^)	−0.155	0.258	−0.093	0.584	−0.245	0.328
VFA (cm^2^)	0.298	0.027	0.207	0.218	0.271	0.277
VFA/SFA	0.289	0.032	0.163	0.335	0.470	0.049
HFC (%)	0.199	0.071	0.048	0.737	0.160	0.339

Data are means ± S.D, BMI: body-mass index, WHR: waist-to-hip ratio, TC: total cholesterol, TG: triglyceride, HDL-c: high-density lipoprotein cholesterol, LDL-c: low-density lipoprotein cholesterol, ALT: alanine aminotransferase, AST: aspartate aminotransferase, γ-GT: γ-glutamyltransferase, SFA: subcutaneous fat area, VFA:visceral fat area, HFC: hepatic fat content.

A stepwise multiple linear regression analysis was performed to determine the contributing factors to serum RBP4 level. Independent variables were those indexes, which were significantly associated with RBP4 in Pearson’s correlation analysis in all subjects. We also performed stepwise multiple linear regression analysis in different gender, respectively. In all subjects, VFA (Standardized Coefficients Beta = 0.357, *p* = 0.019) together with HDL-c (Standardized Coefficients Beta = −0.345, *p* = 0.023) were independent variables significantly associated with serum RBP4. In women, VFA/SFA (Standardized Coefficients Beta = 0.471, *p* = 0.049) was independently associated with serum RBP4, while in men, HDL-c (Standardized Coefficients Beta = −0.315, *p* = 0.040) was variable significantly associated with serum RBP4 (Table 3).

**Table 3:** **Multiple linear regression analysis of RBP4 and Metabolic parameters**

		**Standardized coefficients beta**	**P value**
ALL	VFA	0.357	0.019
	HDL-c	−0.345	0.023
Men	HDL-c	−0.315	0.040
Women	VFA/SFA	0.471	0.049

A step-wise multiple linear regression analysis was performed to determine the contributing factors to serum RBP4 levels. Independent variables included in multivariate regression model were variables whichwere significantly associated with RBP4 in Pearson’s correlation analysis.

### RBP4 and hepatic fat content

**Figure 1:**
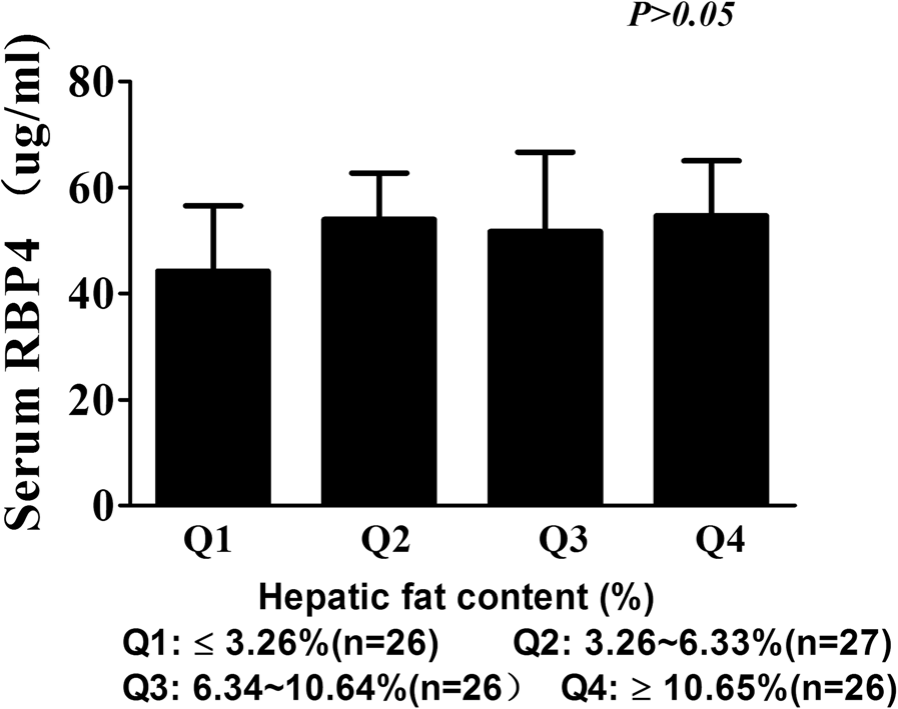
The association between serum RBP4 level and the quartiles of hepatic fat content. When hepatic fat content increased by quartile order, serum RBP4 level was 43.23 ± 1.19, 54.00 ± 8.74, 51.75 ± 1.52, 54.68 ± 8.86 μg/ml respectively. Serum RBP4 is not proportionally increased with hepatic fat content (*p* = 0.298).

The RBP4 concentrations were 43.23 ± 1.19 μg/ml, 54.00 ± 8.74 μg/ml, 51.75 ± 1.52 μg/ml and 54.68 ± 8.86 μg/ml when HFC increased by quartile order, respectively. Although the difference of RBP4 among the four groups was not statistically significant, we found a phenomenon that serum RBP4 level increased when HFC increased from Q1 to Q2, but, it did not increased anymore when HFC increased from Q2 to Q4 (*p* = 0.298, Figure 1, Table 1). So, we predicted that there might be a correlation between RBP4 and HFC when HFC was mildly elevated (less than the median of 6.34%).

**Figure 2:**
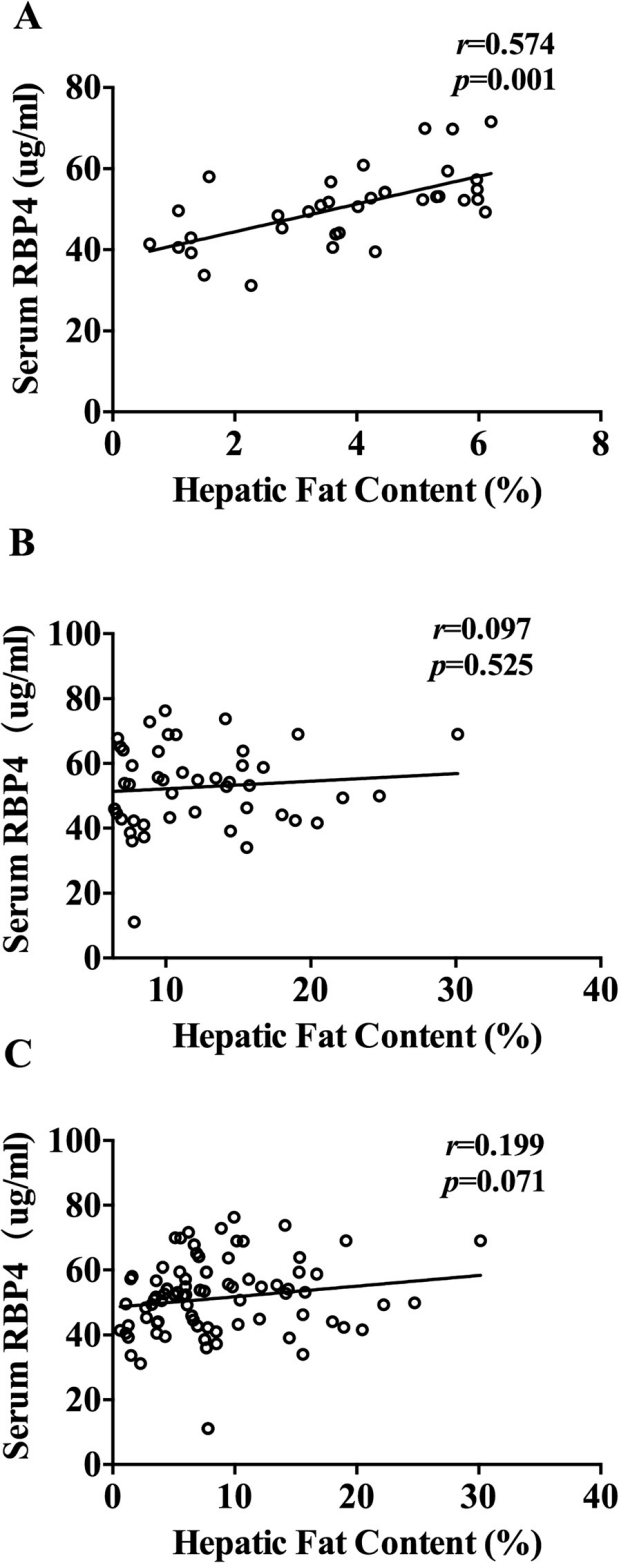
Pearson’s correlation analysis between serum RBP4 level and hepatic fat content. Serum RBP4 level was positively correlated with hepatic fat content when hepatic fat content is less than 6.34%(*r* = 0.574, *p* = 0.001 Figure 2 **A**). However, the association between hepatic fat content and RBP4 no longer existed when hepatic fat content more than 6.34% (r = 0.097, *p* = 0.525 Figure 2 **B**) or all the subjects (r = 0.199, *p* = 0.071 Figure 2 **C**).

In light of the data mentioned above, we analyzed the association between serum RBP4 concentration and HFC in subjects within the first two quartiles of HFC and all subjects, respectively (Figure 2). When HFC within Q1 to Q2 (less than 6.34%), there was a significant positive association between RBP4 and HFC (*r* = 0.574, *p* = 0.001); However, the association between HFC and RBP4 no longer existed when HFC within Q3 to Q4 (more than 6.34%) (*r* = 0.097, *p* = 0.525).

Also, a stepwise multiple linear regression analysis was performed in subjects whose HFC were less than 6.34%, using HFC as a dependent variable, RBP4 as an independent variable together with weight, BMI and waist circumference, which are significantly associated with hepatic fat content. And the results showed that RBP4 was significantly associated with HFC (Table 4), independent of BMI, weight and waist circumference.

**Table 4:** **Multiple linear regression analysis of RBP4 and HFC (When HFC less than 6.34%)**

	**Standardized coefficients beta**	**P value**
RBP4	0.395	0.014
Weight	0.311	0.049

A step-wise multiple linear regression analysis was performed to determine the contributing factors to hepatic fat content. Independent variables included weight, BMI, waist and RBP4.

## Discussion

To our knowledge, this is the first study to examine the relationship between circulating RBP4 and body fat composition, including hepatic fat content, visceral and subcutaneous abdominal fat in a cohort of Chinese NAFLD patients without known diabetes. And we find that circulating RBP4 is positively associated with the visceral fat, but not hepatic fat content or subcutaneous fat, which implied that visceral fat might be a major source of excess circulating RBP4.

Consistent with our findings, relationship of RBP4 and visceral fat was confirmed by many other studies in different populations [18,25-27], which suggest that visceral obesity might play a key role in increasing the circulating RBP4 level. Some intervention studies were conducted to reduce weight of subjects by methods of bariatric surgery or lifestyle modification and adjuvant appetite suppressants, these results showed that circulating RBP4 level decreased markedly, accompanied by reduction in visceral fat mass [28,29]. The change in RBP4 level was significantly correlated with the amount of abdominal visceral fat loss but not associated with the amount of total body fat loss or abdominal subcutaneous fat loss [29]. Mansouri et al. found that exercise significantly reduced serum concentration of RBP4 in the trained diabetic rats, accompanied by decreased RBP4 protein and gene expression in visceral fat tissue. In contrast, exercise had no significant effect on RBP4 protein and gene expression in liver in the trained diabetic rats [30]. These studies confirmed the relationship between RBP4 and visceral fat from the reverse side.

Although some studies suggest that RBP4 associated with HFC [9,19], we don’t find the relationship between HFC and RBP4 in all the subjects. However, they are significantly correlated when HFC is less than 6.34% (the median of HFC). The disagreement with our findings might be explained by the selection of patients with differing severity and duration of liver disease. Some studies showed that RBP4 protein expression was down-regulated in primary human adipocytes by TNF-α [31,32] and IL-1β [32]. In NASH patients, a variety of inflammatory cytokines rise gradually, exerting different influences on RBP4 expression, so the net concentration of circulating RBP4 can’t keep up with HFC. In addition, RBP4 is mainly secreted by liver, excessive fat deposition and severe lesion in the liver may influence hepatic function, which probably lead to decreasing RBP4 secretion. Hence, we conclude that RBP4 may only reflect HFC on the early stage of fatty liver, but not on the moderate-severe stage.

Schina et al. found that in NAFLD patients, serum RBP4 was significantly lower than that in controls, in contrast, RBP4 liver tissue expression was enhanced and correlated with NAFLD histology [24]. RBP4 liver tissue expression and circulating RBP4 level is not synchronous might be another reason of inconsistent relationship of HFC and RBP4.

No uniform diagnosis method might be another reason for conflicting relationship between HFC and RBP4. Those researches believing HFC and RBP4 were significantly related used ultrasonic method to diagnose fatty liver [9,19], while those researches getting different conclusions took liver biopsy to diagnose NAFLD and divided them into different pathological changes [22,23,33]. And they got a negative conclusion about relationship of RBP4 and nonalcoholic fatty liver disease, and RBP4 decreased along with the deterioration of liver lesions [34].

The other reasons for that there is no relationship between HFC and RBP4 might because those studies employed different study populations as well as various laboratory assay methods and kits used to measure serum RBP4. Graham et al. [35] found considerable discrepancy among different assays measuring serum RBP4 in insulin-resistant individuals. Quantitative western blotting is an accurate assay method for measuring RBP4. In this study serum RBP4 was detected by an enzyme immunoassay kit and validated by quantitative Western blotting. So, the data should be fairly accurate.

In the present work, VFA was associated with RBP4 in women while HDL-c was negatively associated with RBP4 in men, so there were gender discrepancy between RBP4 and some metabolic parameters. Higher levels of circulating RBP4 were discovered in men than in women [17,36]. RBP4 level was different between premenopausal and postmenopausal women and varied with pubertal status [37]. These data suggest that sex hormones play an important role in circulating RBP4 level, and this call for further study.

There are several limitations in this study. Firstly, CT scan is used to determine the hepatic fat content, and its diagnostic accuracy is inferior to pathological diagnosis by liver biopsy and 1H Magnetic Resonance Spectroscopy (^1^H MRS). And we found the liver fat content determined by CT was undervalued, hence, some patients might not have NAFLD and still were included in this study due to the diagnostic inferiority of using a CT scan. So, the results come from this study could just be considered as a clue, and the accurate relationships of RBP4 and fat compartments needs further verification by large scale studies and precise method of assessing hepatic fat content, such as liver biopsy or ^1^H MRS. Secondly, our study is a cross-sectional study, which indicate that cause and effect relationships between RBP4 and body fat composition can not be precisely determined. Thirdly, RBP4 must be bound to retinol for efficient secretion from hepatocytes, retinol deficiency reduces serum RBP4. We do not determine retinol serum concentration and therefore are not able to calculate the theoretic saturation of RBP4 with retinol, which may affect the relationship of RBP4 concentration and metabolic parameters.

In conclusion, RBP4 could be a marker of abdominal obesity, but the role of RBP4 in the pathogenesis of NAFLD is not sufficiently elucidated. These findings should be validated with a larger cohort and more accurate assay methods for diagnosing liver lesion, such as liver biopsy.

## Methods

### Participants

Eligible adults (age 18–70 years) were identified and recruited from the outpatient department of Endocrinology of Zhongshan hospital between Nov. 2005 and Apr. 2006 who were diagnosed as fatty liver by ultrasound. Exclusion criteria were alcohol consumption reported by themselves and their families of more than 30 g/d in the case of men and more than 20 g/d in the case of women. Subjects were also excluded if they had known diabetes, hepatitis B or C or other liver diseases, or if they were receiving hypoglycemic or lipid-regulating (statins, fibrates) drugs, or other drugs could impact hepatic fat content four weeks before the time of enrollment, such as silybin, ursodeoxycholic acid, polyene, phosphatidylcholine, vitamin E, some Chinese herbs, etc. Other exclusion factors were diseases affect the metabolic state or other circumstance not suitable to participate in this clinical trial. These details were described in our previous article [36].

### Anthropometric and biochemical measurements

Clinical data such as age, gender, height, weight were collected. Body mass index (BMI) was calculated as the weight in kilograms divided by the square of the height in meters. Waist circumference was measured at the midpoint between the inferior costal margin and the superior border of the iliac crest on the midaxillary line. Waist–hip ratio (WHR) was calculated as waist circumference divided by hip circumference. Blood pressure was measured three times with 5 minutes intervals each time in the seated position with a mercury sphygmomanometer in the morning, and the average of the three blood pressures was used as the final blood pressure.

Data on demographic variables, health status, health behavior, and physical activity were collected using a standardized questionnaire. All participants were required to fast overnight (12 h) before physical examination by trained staff and physicians using standard protocols. Blood samples were drawn after an overnight fast and immediately centrifuged. Serum glucose was measured by the glucose oxidase method. Serum total cholesterol (TC), triglycerides (TG), high-density lipoprotein cholesterol (HDL-c), low-density lipoprotein cholesterol (LDL-c), and liver enzyme levels were determined by enzymatic methods with a Hitachi 7600 analyzer (Hitachi, Ltd. Tokyo, Japan). Serum RBP4 (μg/ml) was measured in duplicate by a sandwich ELISA developed in-house, using affinity chromatography purified polyclonal and monoclonal antibodies generated against recombinant human RBP4 protein. The assay system was subsequently cross validated by Western blot analysis. The intra-assay coefficient of variation was 1.8–7.6% and inter-assay was 3.7–8.85% [36]. The study was approved by the human research ethics committee of Zhongshan hospital, Fudan University and informed consents were obtained from all subjects.

### Determination of hepatic fat content and body fat distribution with calibrated CT

The abdominal adipose tissue areas were quantified by CT scans using a Marconi MX8000 machine (Philips) by the same one experienced doctor. With the subject in a supine position, a 10-mm CT slice scan was acquired at the L4 to L5 level to measure the total abdominal and visceral fat area. Visceral fat area was quantified by delineating the intra-abdominal cavity at the internal aspect of the abdominal and oblique muscle walls surrounding the cavity and the posterior aspect of the vertebral body. The subcutaneous fat area (SFA) was calculated by subtracting the visceral fat area from the total abdominal fat area. Hepatic fat content was evaluated semi-quantitatively by CT scanning as previously described [36].

### Statistical analyses

All statistical analyses were performed using SPSS18.0 (SPSS Inc., Chicago, Illinois, USA). Normally distributed data were expressed as means ± SD. Data that were not normally distributed, as determined using Kolmogorox–Smirnov test, were logarithmically transformed before analysis and expressed as median with range. One-way ANOVA was used for comparisons among groups, and multiple testing was corrected using LSD method (Equal Variances Assumed) or Games-Howell method (Equal Varance not assumed). Pearson’s correlations and multiple stepwise regression analysis were used to examine the association of serum RBP4, HFC, VFA, SFA and other parameters. In all statistical tests, *p* values <0.05 were considered significant.

## Abbreviations

RBP4: Retinol Binding Protein 4
HFC: Hepatic fat content
VFA: Visceral abdominal fat area
SFA: Subcutaneous abdominal fat area
NAFLD: Non-alcoholic fatty liver disease
NASH: Non-alcoholic steatohepatitis
ALT: Alanine aminotransferase
AST: Aspartate aminotransferase
ALP: Alkaline phosphatase
γ-GT: γ-glutamyltranspeptidase
HDL-c: High-density lipoprotein cholesterol
LDL-c: Low-density lipoprotein cholesterol
SBP: Systolic Blood Pressure (SBP)
DBP: Diastolic blood pressure
BMI: Body mass index
WHR: Waist-to-hip ratio
IR: Insulin resistance
TC: Total cholesterol
TG: Triglyceride
